# Erythrocyte Storage Lesion Improvements Mediated by Naringin Screened from Vegetable/Fruit Juice Using Cell Extract and HPLC-MS

**DOI:** 10.1155/2022/7556219

**Published:** 2022-04-26

**Authors:** Yuqi She, Qiong Liu, Xiyue Xiong, Ning Li, Jian Zhang

**Affiliations:** ^1^Department of Blood Transfusion, Xiangya Hospital, Central South University, Changsha 410008, China; ^2^Clinical Laboratory, The First Affiliated Hospital of Hunan Normal University, Hunan Normal University, Changsha 410002, China; ^3^NHC Key Laboratory of Birth Defect for Research and Prevention, Hunan Provincial Maternal and Child Health Care Hospital, Changsha 410008, China; ^4^National Clinical Research Center for Geriatric Disorders, Xiangya Hospital, Central South University, Changsha 410008, China

## Abstract

In blood banking, storage at 4°C for weeks is known to cause damages to erythrocytes, called storage lesions that may later cause transfusion-related adverse events. In previous experiments, we found that vegetable/fruit juices can effectively reduce the storage lesion. Currently, we attempt to analyze the potential bioactive components and test whether the compounds can improve the storage lesions of erythrocytes. Equal portions in wet weight of 20 fresh vegetables and fruits were blended with phosphate buffered solution (PBS), and clear solutions were produced as additive to the packed erythrocytes from consented blood donors at 1 : 10 ratio (ml : gram). The blood samples were stored for 35 days at 4°C, and the supernatants were performed high liquid chromatography–mass spectrometry (HPLC-MS) analysis at 0 days, 14 days, and 35 days. The blood bags supplemented with identified bioactive components were stored in a refrigerator for 35 days, and the morphology, complete blood count (CBC), phosphatidylserine (PS) extroversion, hemolysis, and reactive oxygen species (ROS) levels were measured at the end of storage. Five potential bioactive components from vegetable/fruit juices contributed to the improvements of storage lesion. One of the compounds was unequivocally identified as naringin, and two were tentatively assigned as vitexin 6″-O-malonyl 2″-O-xyloside and luteolin 7-(6″-malonyl neohesperidoside). Naringin alleviated the storage lesion of red blood cells (RBCs) by reducing ROS levels and living cell extraction with HPLC-MS is a simple, reliable, and effective method for screening potential bioactive components.

## 1. Introduction

Blood transfusion is an important life-saving medical procedure, especially for serious wounds, major operations, and severe anemias. Modern day transfusion requires the involvement of blood banking in which fresh blood products are stored for days or weeks to ensure on-demand availability, blood type matching, and time for safety inspections. Between blood collection and transfusion, red blood cells (RBCs) are separated from other blood ingredients and stored at 4°C for 35 to 42 days in different additive solutions (ASs) [1]. However, storage in blood banking leads to more damages of erythrocytes than fresh unstored erythrocytes, which are collectively referred to as storage lesions [2, 3]. They are characterized by changes from a double-concave disc shape to a smooth spherical shape [4], decreased adenosine triphosphate (ATP) and 2,3-diphosphoglycerate (2,3-DPG) content, oxidation injury, microparticle accumulation, and enhanced adhesion [5]. RBCs storage lesions are closely related to the relevant adverse events caused by transfusion, including acute kidney dysfunction, plasma transferring saturation, reduced survival, increase the incidence of postoperative wound infection, multiple organ failure, and deep vein thrombosis [6–10].

Phytochemicals have been proved to play a role in antioxidant, antithrombotic, anti-inflammatory properties, and so on [11]. For example, naringin has cytoprotective effects against apoptosis and oxidation as a result of its reactive oxygen species (ROS) scavenging effects [12–14]. The vegetables and fruits are a reservoir of abundant phytochemicals. They provide more than 5000 phytochemicals, but a large percentage still remains unknown [15]. When RBCs are stored in vitro, the curtailment of response to oxidative stress in physiological condition result in accumulated ROS and oxidizing the enzymes and lipids [16]. Oxidative damage is a critical cause for RBC storage lesions. Modern storage ASs generally do not contain ingredients aimed at inhibiting oxidation and targeted at providing other nutrients except these we known. Modifications of storage solution with a number of single chemicals have reduced oxidative stress and improved the erythrocyte viability [17–19]. Actually, the phytochemicals have stronger antioxidant activity, and no single chemical can replace the combination of natural phytochemicals in fruit and vegetables in antioxidation [20–22]. The reports show that the antioxidant value of 100 g apples is equivalent to 1,500 mg of vitamin C, and the average vitamin C content in fresh apples with the skin is 5.7 mg per 100 g [20]. That is, the antioxidant value of phytochemicals in 100 g apples is 262 times vitamin C. In a previous study, we mixed juice from 20 fruits and vegetables common in daily life and added to AS to test whether it can improve erythrocyte storage lesions. The results suggested that vegetable/fruit juice reduced the apoptosis and hemolysis of erythrocytes stored in vitro (data not shown). However, it is not clear which effective compounds are responsible.

It is interesting but challenging to identify the bioactive substances from hundreds or even thousands of different components. A novel strategy for predicting bioactive components using live cell extraction and high performance liquid chromatography-mass spectrometry (HPLC–MS) analysis was reported [23, 24]. The hypothesis is that when cells are incubated with the extract, the potentially bioactive ingredients will bind to the cells or be absorbed into the cells. Hence, the concentration of potential bioactive components will decrease after the incubation [23, 24]. In order to screen the targeted bioactive substances which can improve the storage lesions of RBCs from vegetables and fruits, we supplement the vegetables and fruits juices into packed RBCs preserved at low temperatures and compare the composition of supernatant in different storage time by ultra-high-performance liquid chromatography-quadrupole time-of-flight mass spectrometry (UPLC-QTOF-MS). Then, whether the identified components can reduce RBCs storage damage was verified.

## 2. Materials and Methods

### 2.1. Preparation of Vegetable/Fruit Juice

Preparation of vegetable/fruit juice was referred to the report of Li et al. [25]. The vegetables and fruits for making the additive were purchased from the local supermarket of Changsha, Hunan, China, and are listed in Table 1. Five grams of each, for a total of 100 g, were mixed with 900 ml of phosphate buffered solution (PBS) at a ratio 1 : 9 (gram : ml). The mixture was blended in a food blender at full speed for 4 minutes, followed by centrifugation at 3000 g for 15 minutes. The supernatant was adjusted to physiological osmotic pressure and sterilized by passing through a 0.22 *μ*m filter.

### 2.2. Preparation of Leukocyte Filtrated RBCs

Nine volunteer donors were consented according to the standard “Whole blood and component donor selection requirements” in China and recruited. Peripheral blood samples (400 ml ± 10%) were collected from each donor with 56 ml of citrate phosphate dextrose adenine (CPDA) anticoagulant. After filtration of leukocytes and separation of the plasma, erythrocytes were suspended in a 100 ml of mannitol-adenine-phosphate (MAP) AS. This study was approved by the Ethics Committee of Xiangya Hospital of Central South University, China (NO.202103042).

### 2.3. Methods

#### 2.3.1. UPLC-QTOF-MS Analysis

Three packed RBCs were randomly selected to perform the UPLC-QTOF-MS. The packed RBCs were mixed thoroughly and then divided into three equal portions. The first and second parts were added vegetable and fruit solution at 1 : 10 ratios (ml : gram). After blending thoroughly, the supernatant of the first portion was separated immediately through centrifugation at low temperatures to prepare the blank control without RBCs. The second portion acting as the experiment group and control group without RBCs were stored in a refrigerator at 4°C for 35 days. After completely mixing the bags, one-milliliter aliquots plasma was taken at the time points of 0 days, 14 days, and 35 days in a laminar flow cabinet. The third part was injected into PBS at 1 : 10 ratios (ml : gram). After blending thoroughly, the supernatant was collected and stored at −80°C, serving as the blank control without vegetables and fruits solution.

A total of 300 *μ*l plasma and 900 *μ*l cold acetonitril (Merck KGaA, Germany) were vortically oscillated for 30 s. After centrifugation at 13000 rpm at 4°C for 10 min, transfer the supernatant to an EP tube and blow dry with nitrogen. Add 300 *μ*l mixture of acetonitril and water (acetonitril and water ratio: 2 : 1) and eddy for 30 s to redissolve the deposit. Centrifuge at 13000 rpm at 4°C for 10 min, and transfer the supernatant to chromatographic sample bottles.

Chromatographic analysis was performed on an Agilent 1290 infinity ultra-performance liquid chromatography (Agilent Technologies, Palo Alto, CA, USA). Data were acquired and processed by an Agilent MassHunter Qualitative Analysis software (V. B.05.00). A ZORBAX SB C18 column (3.0 mm × 100 mm, 1.8-micron particle size) was employed, and the column temperature was set at 35°C. The mobile phase consisted of (A) water with 0.1% formic acid (J&K Chemical, China) and (B) acetonitrile. The gradient elution conditions were 0-1 min, 5% B, 1–3.5 min, 5–60% B, 3.5–6 min, 60–95% B, 6–10 min, and 95% B. The flow rate was 0.4 ml/min, and the injection volume was 5 *μ*l.

The high-resolution MS analysis was performed on an Agilent 6550 QTOF system (Agilent Technologies) equipped with a dual AJS ESI. Data were acquired and analyzed by Agilent Mass Hunter Profinder software (V. B.08.00) and Agilent Mass Profiler Professional software (V. 13.1.1). The main parameters for MS were as follows: gas temperature 250°C, drying N_2_ gas flow rate 14 L/min, nebulizer pressure 35 psig, fragmentor voltage 380 V, skimmer 65 V, sheath gas temperature 350°C, sheath gas flow 11 L/min, capillary voltage 4000 V, and nozzle voltage (Expt) 1000 V. Full-scan data acquisition was performed from *m*/*z* 50 to 1000 in MS scan mode.

#### 2.3.2. Confirm the Potential Bioactive Component

The reference compound naringin (CAS 10236-47-2, Lot: 110722–202116, purity 93.5%) was purchased from National Institutes for Food and Drug Control of China. Under the conditions described above, the vegetable/fruit juices and standard substance naringin diluted in distilled water to obtain 38 ppm, 19 ppm, 9.5 ppm, and 4.75 ppm were performed UPLC-QTOF-MS analysis, respectively. Compare the retention times and MS data of potential bioactive component with that of standard compounds. According to the chromatographic integration, calculate the regression equation and content of naringin in packed RBCs stored at 0 days. The mean value of naringin in the three groups was recorded as c ppm.

#### 2.3.3. Measurements of Storage Lesions

Six packed RBCs resuspension in MAP solution from each donor was divided into two equal portions. Detect the weight (m) and hematoctit (Hct) value of packed RBCs. Naringin was dissolved and diluted into 300 ppm with PBS. The density of the packed RBCs is approximately 1 g/ml. Let us set the volume of naringin solution added into packed RBCs to v ml. The formula used was

(1) $$\begin{array}{c} v\times 300=\left[m\times \left(1-\frac{\text{Hct}}{100}\right)+v\right]\times c. \end{array}$$

Calculate the value of v and add the corresponding volume of naringin solution with content of 300 ppm into the blood bags, making their final concentrations equal to c ppm. The equivalent PBS was injected into the other blood bags for control groups. Mix the blood bags thoroughly and store in a refrigerator at 4°C for 35 days. One-milliliter aliquots were taken at the end phase of preservation after thoroughly mixing the bags in a laminar flow cabinet. The morphologically of RBCs were viewed under the light microscope OLYMPUS CX23. The complete blood count (CBC), extroversion rate of phosphatidylserine (PS) on the surface of RBC, free hemoglobin, and ROS levels were measured at the time point of 35 days and then calculate hemolysis percentage.

A drop of blood was added to the slide, and another slide was pulled to make a wedge blood smear. After drying, stained with the Wright's stain for 4 minutes, washed with running water, dried, and examined on oil immersion with 100x objective. The CBC was measured using standard clinical chemistry methods and reagents (Mindray, Shenzhen). Free hemoglobin in supernatant was determined using the colorimetric technique (Nanjing Jiancheng, Nanjing). The hemolysis rate was calculated as

(2) $$\begin{array}{c} \text{Hemolysis}=\;\frac{\left(100\;–\text{ hematocrit}\right)\;\times \text{ free plasma hemoglobin}\left(\text{g}/\text{dl}\right)}{\text{Total hemoglobin}\left(\text{g}/\text{dl}\right)\;}. \end{array}$$

The extroversion rate of PS to the RBC surface and ROS levels was determined by flow cytometry (BD, New Jersey). A normal control from healthy people was designed. Collect fresh RBCs, and wash with PBS for three times to remove white blood cells, platelets, and plasma thoroughly. The fluorescent value of normal control was used to divide into the negative and positive. The methods were referred to Kuypers et al. and Blasa et al. reports [26, 27]. Briefly, RBCs were adjusted to 1 × 10^6^ cells/ml and incubated with annexinV—FITC (Beyotime, Shanghai) for 15 minutes at room temperature in the dark to detect PS. 20 mmol/l DCFH-DA (Beyotime, Shanghai) was diluted in PBS to obtain a 10 *μ*mol/l working solution. The erythrocyte suspension was incubated at 37°C with 10 *μ*mol/l DCFH-DA for 45 mins away from light. Then, the cells were washed three times and resuspended with 150 *μ*l PBS for the intracellular ROS test. Acquisition and analysis were performed with the Flowjo software (V.X.0.7).

### 2.4. Statistical Analysis

Statistical analysis was performed using the Statistical Package for the Social Sciences (SPSS) computer software (V.20.0). Data are shown as means ± standard deviation (SD). The data were performed using the paired *t* test, Wilcoxon test, or one-way repeated measures ANOVA. *P* values <0.05 were considered statically significant.

## 3. Results

### 3.1. Bioactive Candidates of Vegetables and Fruits

**Figure 1:**
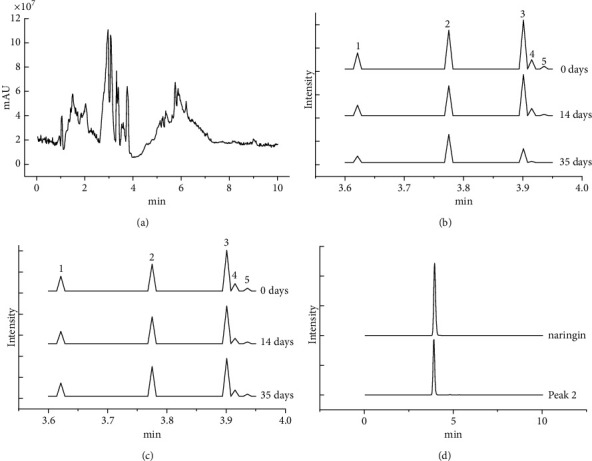
(a) Chromatogram of vegetables and fruits solutions. (b) Chromatograms of five bioactive candidates from vegetables and fruits solutions. Peaks 1, 2, 3, 4, and 5 represent the five bioactive ingredients. All the peak areas decrease gradually from 0 days to 14 days to 35 days during storage. The differences were statistically significant. (c) Chromatograms of five bioactive candidates in blank control without RBCs. Peaks 1, 2, 3, 4, and 5 represent the five bioactive ingredients. All the peak areas have no changes from 0 days to 14 days to 35 days during storage. (d) Chromatograms of naringin and bioactive components of peak 2.

The juice of vegetables and fruits was analyzed by UPLC-Q-TOF-MS. The chromatogram showed that hundreds of peaks were detected in vegetables and fruits under the present chromatographic conditions (Figure 1(a)). Compared with blank control without vegetables and fruits, 35 peaks area in experiment groups have significant differences (data not shown, *P* < 0.05). Among them, there were five (peaks 1–5) peaks area decreasing gradually after the extracts were incubated with packed RBCs from 0 days to 35 days (Figure 1(b)). One-way repeated measures ANOVA showed that there were statistical differences in these five peaks area at 0 days, 14 days, and 35 days (*P*=0.012, 0.001, 0.049, 0.047, 0.044, respectively). Meanwhile, no remarkable changes of the five peaks area had been observed in the control group without RBCs stored at 4°C for 35 days (Figure 1(c)), meaning that the five bioactive components had no obvious degradation during storage for 35 days. The five peaks can be found in vegetable and fruit juices but not in blank control without vegetables and fruits. These results suggested that the five components in extracts of vegetables and fruits have the interactions with packed RBCs. These cell-interacting compounds could be regarded as the potential bioactive candidates of vegetables and fruits for improving the storage lesions of RBCs.

### 3.2. Confirm the Potential Bioactive Component

**Figure 2:**
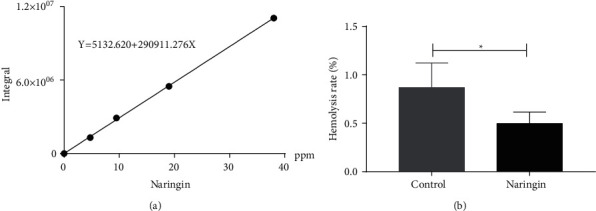
(a) Regression equation is *Y*=5132.620 + 290911.276X. The *X*-axis is naringin content with 4.75 ppm, 9.5 ppm, 19 ppm, and 38 ppm and *Y*-axis represents the corresponding chromatographic integral. (b) Hemolysis of packed RBCs at 35 days. ^*∗*^ signifies *p* < 0.05 vs control group.

The five substances are identified by comparing the retention time and mass spectrometric data of standard substance or referring to the literatures. The MS data of five area-changing peaks are presented in Table 2. The potential compound of peaks 2 was definitively identified as naringin by comparison of retention times and MS data with that of standard compounds (Figure 1(d)). Peaks 3 and 5 were transiently assumed to be vitexin 6″-O-malonyl 2″-O-xyloside and luteolin 7-(6″-malonylneohesperidoside) by the comparison of the mass data with that of literatures [28–30]. However, for peaks 1 and 4, their identities were still unknown and only molecular formula was calculated. According to the chromatographic integration of 38 ppm, 19 ppm, 9.5 ppm, and 4.75 ppm naringin, the regression equation is *Y* = 5132.620 + 290911.276X (*F* = 1219.615, *P*=0.001, *R*^2^ = 0.998) (Figure 2(a)). The content of naringin in packed RBCs is about 12 ppm.

### 3.3. Morphological Analysis of Erythrocytes

**Figure 3:**
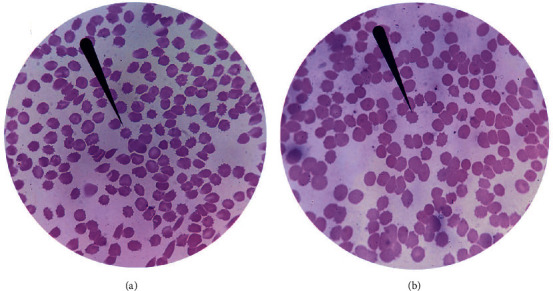
Storage lesion of packed erythrocytes without (a) or with (b) naringin (×1000). The RBCs were performed Wright's stain. RBCs morphology including discocytes, echinocytes, sphero-echinocytes, and spherocytes was observed.

To investigate the possible protective role of naringin on RBCs, the morphology of erythrocytes stored for 35 days was observed under light microscope. As shown in Figure 3, the both groups were undergoing the storage lesion. RBCs morphology including discocytes, echinocytes, sphero-echinocytes, and spherocytes was observed. It seems that the experimental group had less abnormal RBCs than the control group, suggesting that RBCs in the treatment group suffer from less serious damage.

### 3.4. ROS of RBCs at 35 Days

**Figure 4:**
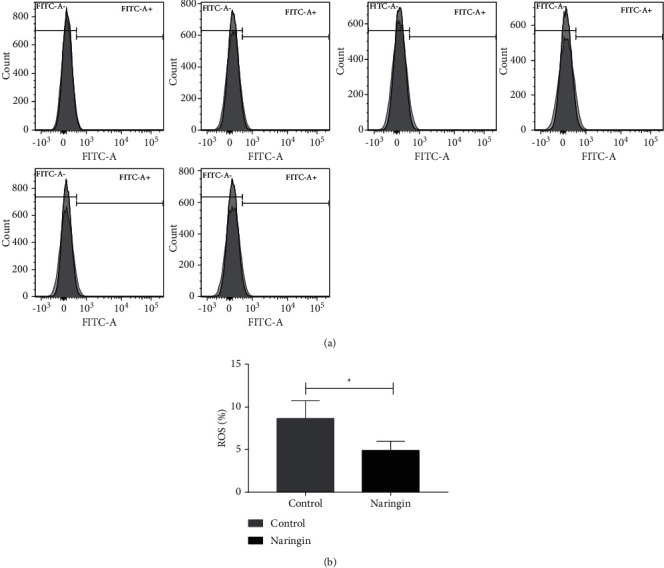
(a) Flow cytometry histograms of ROS assays in packed RBCs. The RBCs were stored at 4°C for 35 days with naringin solution or equal volume of PBS. (b) ROS of packed RBCs at 35 days. ^*∗*^ signifies *p* < 0.05 vs control group.

In vitro, the reduced properties of antioxidant stress led to the accumulated ROS and contributed to the RBCs storage lesion. ROS was investigated by flow cytometer with DCFH-DA. The histogram plots of flow cytometer analyses of ROS from different derived RBCs are shown in Figure 4(a). The ROS in the experimental group was lower than those in the control group (*P*=0.028) (Figure 4(b)).

### 3.5. PS Extroversion Rate of RBCs after 35 Days of Storage

**Figure 5:**
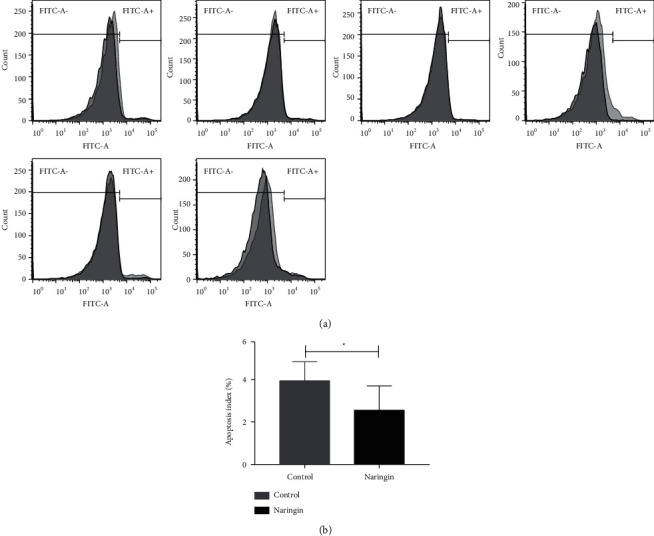
(a) Flow cytometry histograms of apoptosis assays in packed RBCs. The RBCs were stored at 4°C for 35 days with naringin solution or equal volume of PBS. (b) Apoptosis of packed RBCs at 35 days. ^*∗*^ signifies *p* < 0.05 vs control group.

PS is distributed in the inner leaflet of the plasma membrane under normal condition. In apoptotic cells, the membrane PS is translocated from the inner to the outer leaflet of the plasma membrane, thereby exposing PS to the external cellular environment [31]. The PS extroversion serves as a signal for eryptosis. PS was investigated by flow cytometer at the end of storage. The histogram plots of flow cytometer analyses of the PS extroversion rate from different derived RBCs are shown in Figure 5(a). The PS extroversion rate in the experimental group was lower than those in the control group (*P*=0.028) (Figure 5(b)).

### 3.6. Hemolysis of RBCs at 35 Days

Hemolysis suggests that the structural integrity of RBCs has been destroyed and causes lysis. The hemolysis rate of RBCs in experimental group was lower than those in the control group at 35 days (*P*=0.046) (Figure 2(b)).

## 4. Discussion

The average lifespan of human erythrocytes is about 120 days. After collection, erythrocytes in the blood bag are a mixture of cells with residual lifespan ranging from 0 to 120 days. It is expected a theoretical loss of 1/120 of cells per day at the normal temperature or a loss of about 29% after 35 days. The standard storage condition is to resuspend erythrocytes in AS and to lower the temperature to 4°C. Under this kind of conditions, the preservation of erythrocytes is up to the criteria that the hemolysis is less than 1% and at least 75% of the red cells survive 24 hours after transfusion [32]. However, the storage lesions are still inevitable and able to result in a number of clinical sequelae [16].

Vegetables and fruits are universally promoted as healthy. The Dietary Guidelines for Americans 2015–2020 recommend you make one-half of your plate fruits and vegetables [33]. One of the primary reasons is that vegetables and fruits contain a number of phytochemicals such as flavonoids, lignans, saponins, and peptides. They exert functions including antioxidant, nutrition, anti-inflammatory, anti-apoptotic, and so on [34–36]. In our previous studies, vegetables and fruits juices were found to improve the storage lesion of RBCs. However, the potential bioactive components were unclear. We added vegetable and fruit juices into packed RBCs at the ratios of 1 : 5, 1 : 10, and 1 : 20 (ml : gram) and detected the ATP, 2.3-DPG, Na^+^-K^+^ ATPase, PS, and hemolysis after 35 days of storage. The results suggested that only PS exposure and hemolysis were significantly different against the control, and the ratio of 1 : 10 was the lowest levels among the three groups (data not shown). Hence, the ratio 1 : 10 was selected to screen the bioactive candidates, and PS exposure and hemolysis were chosen as two of the main parameters of storage lesions.

The conventional method for studying bioactive components was burdensome, since there may be hundreds, even thousands of compounds to isolate, purify, and evaluate the bioactivity. Recently, the application of living cell extraction with HPLC-MS for screening the potential components has attracted much attention [37–39]. The cell extraction method was simple and reliable to screen the bioactive substance. The packed RBCs stored in refrigerator can survive for a long time in a relatively simple culture environment. Meanwhile, the AS supply RBCs the energy source to transfer or absorb the bioactive components. Both make RBCs a good model for live cell extraction. Hence, in this research, we attempt to analyze the potential bioactive components using live cell extraction couple with HPLC-MS. When stored at 4°C, the metabolisms of RBCs just slow down but do not completely cease [40]. Therefore, in order to be more accordant with the practical situations, the packed RBCs and extracts of vegetables and fruits were incubated at 4°C, not 37°C, compared with the original method. The period was also prolonged to 35 days. We monitored the variations of composition in the supernatant at 0 days, 14 days, and 35 days during storage. There were at least five bioactive components in vegetables and fruits decline gradually. This manner of reduction dependent on the time seems as nonphysical adsorption, but bio-selective binding. Because of the long-term storage, the absorbed components were highly likely to be synthesized or converted into other substances. Hence, it was hardly to track them in RBCs. One of the five potential compounds was unequivocally identified as naringin, and two were tentatively assigned as vitexin 6″-O-malonyl 2″-O-xyloside and luteolin 7-(6″-malonyl neohesperidoside). The remaining two were still unknown.

Naringin is a bio-flavonoid that is derived from grapefruit and related citrus species and has been reported to possess potent antioxidant/free radical scavenging properties [41]. In metabolic syndrome, apart from alleviating syndrome directly, naringin also restrain the progress of metabolic syndrome through inhibition of oxidative stress [42]. Naringin significantly retarded the progression of fibrosis in a diabetic heart through its antioxidant actions [43]. In vitro model of erythrocytes aging induced by paclitaxel, naringin inhibited the aging by lessening the oxidative stress [12]. When RBCs were stored ex vivo at low temperature, the oxidative stress initiated the damages. Lack of nutrients, substrates, and accumulation of metabolic waste products aggravated the lesions. Meanwhile, loss of biochemical countermeasures that was functional in vivo resulted in the RBCs death [16]. The data showed that naringin significantly reduced packed erythrocytes the PS extroversion, hemolysis, and ROS levels. It meant that naringin improved RBCs storage lesions by attenuating ROS levels.

Vitamin C and E are two of the most common antioxidants. However, they have not changed as shown for naringin. Vitamin E is fat soluble, and it cannot be extracted when preparing vegetable and fruit juices with PBS. Vitamin C is water soluble. However, RBCs have receptors only for oxidized vitamin C, that is, RBCs can only absorb oxidized vitamin C, but not reduced vitamin C [44]. Second, RBCs stored at 4°C metabolize 1/10 slower than those stored at 25°C. The rates of glycolysis and ATP consumption fall by 10–15% per degree Celsius [40]. The decrease in metabolic rate affects vitamin C absorption. Finally, vitamin C is unstable, and significant degradation can occur after 30 minutes of storage at room temperature or 14 days storage at −70°C [45]. In the preparation of vegetable and fruit juice, high-speed centrifugation during cell wall disruption leads to the rise of temperature and accelerate the degradation of vitamin C.

Whether a compound can be used clinically, toxicological tests and safety evaluations are the primary factors to be considered. The RBCs stored in vitro are eventually transfused back into the body. Therefore, if safety is not up to criterion, even though the curative effects are very good, the application prospects look pretty gloom. Studies showed that the oral single dose of 16 g/kg of naringin did not produce acute oral toxicity in Sprague–Dawley (SD) rats, and in the 13-week subchronic oral toxicity study, daily doses of 50, 250, and 1250 mg/kg of naringin were well tolerated and did not cause either lethality or toxic clinical symptoms [46]. The NOAEL (no-observed-adverse-effect-level) of naringin is proposed to be greater than 1250 mg/kg/day following daily oral administrations to SD rats for six months (corresponding to 12 g for a 60 kg human) [47]. The cytotoxicity assay showed that naringin had no toxicity to VERO and MDCK cells at concentrations ranging from 125 to 750 *μ*g/mL [48]. Hence, naringin is generally considered to be a relatively harmless or nontoxic substance. Although naringin, unlike the N-acetyl-L-cysteine (NAC), another ROS scavenger [49], is not yet widely used in clinical practices, low concentrations of naringin for erythrocyte preservation should be a safe and feasible method.

There were some limitations in our study. The LC-MS method has many advantages, including improving the sensitivity and specificity, boosting the efficiency, and being able to identify the structure of compounds. However, there are some disadvantages to this approach. Some small nonpolar compounds such as aldehydes and ketones, amino acids, a small molecule containing a carboxyl group, unstable compounds, and proteins are not suitable for LC-MS. Some trace materials with extremely low levels cannot be measured by LC-MS. When multiple components are analyzed simultaneously, it is difficult to meet all the analysis requirements under certain condition due to the different mass spectrum response, linear range, and sensitivity of individual molecules. The matrix effect results in either ion suppression or ion enhancement. Carry-over effect influences the accuracy of compounds at low levels. Pitfalls in the accuracy of LC-MS methods still arise from in-source fragmentation of conjugate and isomers derivatives of a target analyte [50, 51]. Therefore, not all the bioactive candidates can be detected by LC-MS because of the inherent disadvantages. This may be partly explains why there are hundreds of components in fruit and vegetable juices, but only five bioactive candidates were exploited. Second, because of the method cell extract with HPLC-MS focusing on the compounds with decreased levels, there is a limitation to evaluate these increasing ingredients such as lactic dehydrogenase (LDH), lactic acid, which are important indicators of storage lesion [52]. Besides, as a powerful antioxidant, naringin improves RBC storage lesion possibly by affecting other parameters of redox in addition to ROS. Hence, the protective effect of naringin requires further analysis of more parameters of storage lesion.

## 5. Conclusions

The method of live cell extraction coupled with HPLC-MS is feasible, rapid, and useful to screen potential bioactive components. Five potential bioactive components from vegetable/fruit juices contributed to the improvements of storage lesion. Naringin screened from vegetables and fruits significantly improves the lesions of erythrocytes by reducing ROS levels. Another two were tentatively assigned as vitexin 6″-O-malonyl 2″-O-xyloside and luteolin 7-(6″-malonyl neohesperidoside). The remaining two were still unclear.

## Figures and Tables

**Table 1:** Components of vegetable/fruit juice.

Components of vegetable/fruit juice	Weight (g)	Components of vegetable/fruit juice	Weight (g)
White gourd	5	Apple	5
Tomato	5	Watermelon	5
Cucumber	5	Hami melon	5
Snow pear	5	Grape	5
Towel gourd	5	Banana	5
Pumpkin	5	Plum	5
Carrot	5	Peach	5
Chinese cabbage	5	Pitaya	5
Celery	5	Kiwi fruit	5
Pomelo	5	Orange	5

**Table 2:** MS data of five bioactive candidates of vegetables and fruits.

Peak no.	Rt (min)	[M + H]^+^ (*m*/*z*)	Molecular formula	Identity
1	3.625	646.7998	C_22_NO_13_S_5_	Unknown
2	3.782	581.1865	C_27_H_32_O_14_	Naringin
3	3.903	651.1556	C_29_H_30_O_17_	Vitexin 6″-O-malonyl 2″-O-xyloside
4	3.904	652.1594	C_23_H_25_N_9_O_14_	Unknown
5	3.931	681.1661	C_30_H_32_O_18_	Luteolin 7-(6″-malonylneohesperidoside)

## Data Availability

Data used to support the findings of this study are available on request to the corresponding author (zhangjxy0512@qq.com).
